# Hypertension, Antihypertensive Use and the Delayed‐Onset of Huntington's Disease

**DOI:** 10.1002/mds.27976

**Published:** 2020-02-04

**Authors:** Jessica J. Steventon, Anne E. Rosser, Emma Hart, Kevin Murphy

**Affiliations:** ^1^ Cardiff University Brain Research Imaging Centre (CUBRIC), School of Physics and Astronomy, Maindy Road Cardiff University Cardiff Wales UK; ^2^ Neuroscience and Mental Health Research Institute and Brain Research and Intracerebral Neurotherapeutic (BRAIN) unit, School of Medicine Cardiff University Cardiff Wales UK; ^3^ Brain Repair Group, School of Biosciences Cardiff University Cardiff Wales UK; ^4^ Bristol Heart Institute (BHI), Clinical Research and Imaging Centre, School of Physiology, Pharmacology and Neuroscience Bristol University Bristol UK

**Keywords:** blood pressure, cardiovascular risk, hypertension, neurodegeneration

## Abstract

**Background:**

Hypertension is a modifiable cardiovascular risk factor implicated in neurodegeneration and dementia risk. In Huntington's disease, a monogenic neurodegenerative disease, autonomic and vascular abnormalities have been reported. This study's objective was to examine the relationship between hypertension and disease severity and progression in Huntington's disease.

**Methods:**

Using longitudinal data from the largest worldwide observational study of Huntington's disease (n = 14,534), we assessed the relationship between hypertension, disease severity, and rate of clinical progression in Huntington's disease mutation carriers. Propensity score matching was used to statistically match normotensive and hypertensive participants for age, sex, body mass index, ethnicity, and CAG length.

**Results:**

Huntington's disease patients had a lower prevalence of hypertension compared with age‐matched gene‐negative controls. Huntington's disease patients with hypertension had worse cognitive function, a higher depression score, and more marked motor progression over time compared with Huntington's disease patients without hypertension. However, hypertensive patients taking antihypertensive medication had less motor, cognitive, and functional impairment than Huntington's disease patients with untreated hypertension and a later age of clinical onset compared with untreated hypertensive patients and normotensive individuals with Huntington's disease.

**Conclusions:**

We report the novel finding that hypertension and antihypertensive medication use are associated with altered disease severity, progression, and clinical onset in patients with Huntington's disease. These findings have implications for the management of hypertension in Huntington's disease and suggest that prospective studies of the symptomatic or disease‐modifying potential of antihypertensives in neurodegenerative diseases are warranted. © 2020 The Authors. *Movement Disorders* published by Wiley Periodicals, Inc. on behalf of International Parkinson and Movement Disorder Society.

Huntington's disease (HD) is a neurodegenerative disorder caused by the expansion of a CAG repeat sequence in exon 1 in the Huntingtin gene,^1^, ^2^ leading to the expression of mutant huntingtin (mHtt) protein containing an expanded polyglutamine stretch. The earliest and most marked brain change is striatal atrophy, although more widespread changes are increasingly seen with disease progression.^3^, ^4^, ^5^ Alongside the characteristic triad of cognitive, behavioral, and movement deficits,^6^ a range of symptoms suggestive of autonomic nervous system (ANS) dysfunction have been shown to exist in patients with HD. For example, excessive sweating, micturition difficulties, orthostatic intolerance, sexual dysfunction, gastrointestinal problems, and tachycardia have been reported in patients across disease stages, including premanifest gene carriers,^7^, ^8^, ^9^, ^10^ suggesting that autonomic symptoms contribute to the clinical phenotype of HD and disease burden.

The autonomic nervous system plays a fundamental role in modulating cardiovascular functions, including the control of blood pressure.^11^ Hypertension and disturbances in blood pressure regulation have been associated with Parkinson's disease^12^, ^13^, ^14^ and Alzheimer's disease,^15^ whereas peptides of the renin‐angiotensin system, a prominent therapeutic target in hypertension, have been implicated in the pathophysiology of neurodegenerative diseases^16^ including HD, suggesting that hypertension impacts the neurodegenerative process.

Despite this, the functioning of the ANS has been relatively underexplored in HD. Neuronal inclusion bodies of mHtt occur throughout the hypothalamus,^17^ which is a key hub for autonomic regulation. A handful of studies to date have shown evidence of ANS dysfunction,^18^, ^19^, ^20^ with some evidence that both the sympathetic and parasympathetic branches of the ANS are affected,^19^, ^21^ whereas others have shown evidence of intact or even increased sympathetic activity.^22^ Kobal et al^20^ carried out a battery of cardiovascular autonomic testing in HD patients and found an effect of HD disease stage on autonomic functioning. Premanifest HD gene carriers were found to have a higher Valsalva ratio and higher low‐frequency power of the heart rate variability spectrum compared with controls, whereas early‐stage manifest HD patients displayed a higher sympathovagal ratio, which together suggest higher sympathetic activity in early stages of the disease. In contrast, HD patients with more advanced stages of the disease were found to have a smaller increase in diastolic blood pressure during a handgrip along with a decreased respiratory and orthostatic ratio.^20^

Although the most prominent symptoms of HD are related to the central nervous system, mHtt is ubiquitously expressed in human tissue,^2^ prompting more attention to be paid to the possible involvement of non‐CNS tissues and organs.^23^, ^24^, ^25^ The vascular contribution to HD pathology is increasingly recognized^4^, ^26^ and thought to involve multiple pathogenic pathways.^27^, ^28^, ^29^ Nevertheless, little is known about the prevalence and clinical effect of cardiovascular risk factors such as hypertension on patients with HD. This is important, as evidence shows that both genetic and environmental factors can modify the age at onset and disease course in HD.^30^, ^31^ Previous work using the Registry database, a European subset of the worldwide Enroll‐HD data set,^32^ showed a paradoxical delay in the clinical onset of HD in patients with hypertension, although the potentially confounding effects of age, sex, and body mass index (BMI)^33^, ^34^, ^35^ were not accounted for. Here, we used data from Enroll‐HD, which is the global follow‐on study from Registry and comprises more extensive and more stringently monitored data. We statistically matched normotensive HD patients with HD patients with hypertension based on age, sex, BMI, ethnicity, and polyglutamine expansion length to comprehensively characterize the effect of hypertension on HD symptom severity and progression while accounting for confounding variables.

## Methods

### Participants

Enroll‐HD is a multicenter longitudinal observational study designed to facilitate clinical research in HD. Core data sets are collected annually. We retrieved the fourth periodic version of the Enroll‐HD database (version 2.0), which contained observational data from 15,301 participants (55.6% female, 3539 premanifest HD, 8043 manifest HD, 3629 gene‐negative and/or family controls at baseline; 50,452 visits in total) and integrated longitudinal data from 5355 individuals who had previously participated in the Registry study. Data were monitored for quality and accuracy using a risk‐based monitoring approach.

All participating sites were required to obtain and maintain local ethics committee approval, and all participants gave signed informed consent for their data to be included in accordance with the Declaration of Helsinki.

Participants aged younger than 18 years old at their baseline visit were excluded. HD participants with a CAG length recorded as >70 were excluded, as the precise length was not available. Manifest HD status was determined by the rater and expressed as a diagnostic confidence level of 4, indicating unequivocal motor signs of HD (≥99% confidence).

### Hypertension Status

Participants with a current diagnosis of essential (primary) hypertension (I10) were coded using the International Classification of Diseases (10th revision), which excludes hypertension complicating pregnancy, neonatal hypertension, primary pulmonary hypertension, and primary and secondary hypertension involving vessels of the brain or eye and those with comorbid heart or kidney disease. Hypertensive participants with a history of antihypertensive medication use but whose treatment was not ongoing were excluded from the analyses focused on medication effects. Antihypertensive medication was coded using the Anatomical Therapeutic Chemical classification system (see Supplementary information).

### Clinical Outcome Measures

Performance on motor, functional, behavioral, and cognitive subdomains of the Enroll‐HD assessment were used as a measure of disease severity and progression. Full details can be found in the study protocol at https://www.enroll-hd.org/.

The measures of interest were total motor score (TMS), depression and anxiety score on the Hospital Anxiety and Depression Scale, total functional capacity (TFC), and all the Unified Huntington's Disease Rating Scale cognitive subdomains.^36^ TFC is a measure of capacity to work, handle finances, perform chores and self‐care, and live independently, and the scale ranges from 13 (normal) to 0 (severe disability).

The 4 cognitive subtests were: Stroop (word reading, color naming, and interference), Trail Making (parts A and B), Verbal Fluency (categories and letter), and the Symbol Digit Modalities Test. Additional details can be found in the Supplementary Information.

### Statistical Analysis

Statistical analysis was performed using the open‐source software RStudio (version 1.1.463), and the code used can be accessed at http://doi.org/10.17035/d.2019.0079578885 .

Age, BMI, sex, and ethnicity were identified as a priori confounding variables.^35^, ^37^, ^38^ Propensity score matching with nearest neighbor matching was used to match (2:1 ratio) the 2 groups of interest and adjust for confounds.^39^, ^40^ Propensity scores were calculated using age, BMI, sex, and ethnicity, along with CAG length for HD‐specific analysis; the resulting sample size and demographics of included patients for each analysis are shown in Table 1. Supplementary Tables 1–3 show the demographics after stratifying for antihypertensive medication use. Following matching, if the standardized mean difference (SMD) between groups was greater than 0.10 or *P* < 0.01, the variable was demeaned and added as a covariate in the regression model to remove residual confounding bias.^41^

**Table 1:** Demographics of included participants in the 7 separate statistical analyses conducted

	n	Age	Male, n (%)	BMI	CAG length	Ethnicity (white), n (%)	n	Age	Male, n (%)	BMI	CAG length	Ethnicity (white), n (%)	Age	Sex	BMI	CAG length	Ethnicity (white), n (%)
	Gene‐negative controls						HD						SMD				
(1) Prevalence	3616	46.9 ± 14.7	1409 (39.0)	27.9 ± 6.2	20.1 (3.5)	3248 (89.8)	7233	46.7 ± 13.5	2799 (38.7)	27.0 ± 5.2	43.6 (3.7)	6643 (91.9)	0.02	0.01	0.14	NA	0.14
	Normotensive HD						Hypertensive HD						SMD				
(2) Age at onset^a^	2410	60.5 ± 9.9	1221 (50.7)	26.2 ± 5.2	42.4 ± 2.1	2290 (95.0)	1205	61.2 ± 10.6	608 (50.5)	26.8 ± 5.3	42.2 ± 2.2	1138 (94.4)	0.07	0.004	0.1	0.03	0.06
(3) Disease severity	3032	58.7 ± 10.8	1516 (50.0)	26.9 ± 5.7	42.0 ± 2.2	2885 (95.2)	1516	59.6 ± 11.5	766 (50.5)	27.4 ± 5.7	41.9 ± 2.3	1430 (94.3)	0.08	0.01	0.09	0.03	0.08
(4) Disease progression	4507	55.3 ± 11.7	2193 (48.7)	26.4 ± 5.4	42.4 ± 2.5	4235 (94.0)	1521	59.6 ± 11.5	752 (49.4)	27.5 ± 5.7	41.9 ± 2.3	1435 (94.3)	0.37	0.02	0.19	0.21	0.04
	Untreated hypertensive (HD only)						Treated hypertensives (HD only)						SMD				
(5) Age at onset^a^	297	60.5 ± 9.9	158 (53.2)	26.0 ± 4.9	42.4 ± 2.4	283 (95.3)	908	61.5 ± 10.5	450 (49.6)	27.0 ± 5.4	42.1 ± 2.2	855 (94.2)	0.07	0.05	0.13	0.07	0.12
(6) Disease severity	424	58.41 ± 11.95	202 (47.6)	27.0 ± 5.8	42.2 ± 2.4	1076 (94.1)	1163	59.9 ± 11.4	597 (51.3)	27.5 ± 5.6	41.9 ± 2.2	354 (95.2)	0.09	0.05	0.06	0.09	0.093
(7) Disease progression	372	58.4 ± 11.83	192 (51.6)	27.1 ± 4.9	42.1 ± 2.4	354 (95.2)	1149	59.9 ± 11.4	560 (48.7)	27.6 ± 5.6	41.9 ± 2.2	1091 (94.1)	0.28	0.05	0.14	0.17	0.08

SMD, standardized mean difference; BMI, body mass index. Covariates were included in the analyses when SMD > 0.1.

The summary of balance after propensity matching (2:1 ratio, nearest neighbor matching) is shown. Gray‐shaded rows show the groups used for matching for the various analyses.

a Age‐at‐onset analysis was conducted in manifest HD participants only.

The prevalence of hypertension in HD participants compared with matched controls was statistically tested using a logistic regression, with confounding factors included to determine potential independent risk factors for hypertension. Where significant, a Wald test assessed each variable's contribution to the model.

In all analyses except age at onset, premanifest and manifest HD participants were included. Multiple comparisons were adjusted using the false‐discovery rate (*q* = 0.05).^42^ To reduce the dimensionality of the cognitive scores, we performed a principal components analysis (n = 4578) on the 8 standardized and transformed cognitive subdomain scores to derive a summary statistic that could capture most of the variation. The first principal component (PC1) accounted for 60.01% of the variation and was used as a dependent variable.

A linear regression analysis examined the effect of hypertension (normotensive vs hypertensive) and antihypertensive medication status (levels: normotensive, treated hypertensive, untreated hypertensive) on baseline measures. Post hoc pairwise comparisons applied a *P* = 0.05 Tukey‐adjusted significance level.

To examine the interaction between hypertension and clinical progression over time, we applied linear mixed‐effects models using the lme4 package with fixed and random terms to account for the correlation between the repeated measurements for each individual and visit day as the time variable.

## Results

Group demographics for each analysis after propensity matching are shown in Table 1. For the longitudinal analysis, the mean visit day was 468 ± 464 days, with a range from 0 to 2184 days (see Supplementary Fig. 1).

### Demographics of the Cohort by Hypertension Diagnosis

Table 2 shows the demographics of the Enroll‐HD database; 2248 participants (15.5%) had a diagnosis of essential hypertension, and of these, 1697 (75.5%) were currently prescribed antihypertension medication. Hypertensive participants were older, had a higher BMI, a lower CAG repeat length, and a lower female:male ratio. Manifest HD participants were older and had a lower BMI compared with premanifest HD and control participants, and premanifest HD participants were younger with a lower BMI than control participants (all *P* < 0.0001), justifying the use of propensity matching to account for confounding variables. Across the course of the study there was no difference in the frequency of death between normotensive and hypertensive HD participants (*P* = 0.997).

**Table 2:** Demographics at entry point (baseline visit) based on HD and hypertension status after exclusion criteria applied and prior to propensity matching

	Premanifest HD			Manifest HD			Controls			Hypertension		HD status
	(n = 3503)			(n = 7409)			(n = 3622)					
	Normo	Hyper		Normo		Hyper	Normo	Hyper	*P*			*P*
Baseline, n	3243	260		6196		1213	2921	701				
Female, n (%)	1942 (59.9)	145 (55.8)		3231 (52.1)		602 (49.6)	1824 (62.4)	389 (55.5)		**0.0002**		**<2.2 × 10** ^**−16**^
CAG, mean ± SD	42.5 ± 2.8	40.8 ± 2.0		44.5 ± 4.0		42.2 ± 2.3				**<0.0001**		
Age, mean ± SD	38.7 ± 11.4	51.9 ± 12.5		51.5 ± 12.2		61.2 ± 10.6	44.2 ± 14.0	58.6 ± 11.5		**<0.0001**		**<0.0001**
BMI, mean ± SD	25.9 ± 5.3	29.8 ± 6.0		24.4 ± 4.8		26.7 ± 5.3	27.1 ± 5.9	31.2 ± 6.6		**<0.0001**		**<0.0001**
History of tobacco use, %	43.6	44.3		48.9		48.4	42.1	46.3		0.24		**<0.0001**
Region, n (%)												
North America	1170 (36.1)	101 (38.8)		1627 (26.3)		372 (30.7)	1402 (48.0)	398 (56.8)		0.67		**<0.0001**
Europe	1871 (57.7)	142 (54.6)		4361 (70.4)		784 (64.6)	1407 (48.2)	280 (39.9)				
Australasia	195 (6)	17 (6.5)		158 (2.6)		41 (3.4)	88 (3.0)	15 (2.1)				
Latin America	7 (0.2)	0		50 (0.8)		16 (1.3)	0 (0)	8 (1.1)				
Ethnicity, n (%)										0.82		**<0.0001**
White	3030 (93.4)	244 (93.8)		5823 (94)		1146 (94.5)	2626 (89.9)	628 (89.6)				
American Black	13 (0.4)	2 (0.8)		61 (1)		12 (1.0)	26 (0.9)	11 (1.6)				
Hispanic/Latino	62 (1.9)	2 (0.8)		109 (1.8)		23 (1.9)	80 (2.7)	28 (4.0)				
American Indian	49 (1.5)	6 (2.3)		92 (1.5)		17 (1.4)	51 (1.7)	9 (1.3)				
Asian	15 (0.5)	1 (0.4)		19 (0.3)		3 (0.2)	63 (2.2)	13 (1.9)				
Mixed	52 (1.6)	1 (0.4)		46 (0.7)		8 (0.7)	43 (1.5)	7 (1.0)				
Other	22 (0.7)	4 (1.5)		42 (0.7)		4 (0.3)	32 (1.1)	5 (0.7)				
ISCED education level, n (%)										0.08		**0.0009**
0	1 (0)	0 (0)		23 (0.4)		3 (0.2)	2 (0.1)	6 (0.9)				
1	23 (0.7)	3 (1.2)		267 (4.3)		78 (6.4)	77 (2.6)	30 (4.3)				
2	330 (10.2)	37 (14.2)		1199 (19.4)		239 (19.7)	258 (8.8)	75 (10.7)				
3	836 (25.8)	80 (30.8)		2072 (33.4)		439 (36.2)	803 (27.5)	205 (29.2)				
4	704 (21.7)	57 (21.9)		1065 (17.2)		181 (14.9)	631 (21.6)	144 (20.5)				
5	1235 (38.1)	78 (30)		1396 (22.5)		245 (20.2)	1054 (36.1)	224 (32.0)				
6	105 (3.2)	3 (1.2)		139 (2.2)		22 (1.8)	86 (2.9)	16 (2.3)				
Comorbidities/concomitant^a^												
Comorbidity,^a^ n (%)	2627 (81.0)	241 (92.7)		5480 (88.4)		1154 (95.1)	2163 (74.0)	632 (90.2)		**<0.0001**		**<0.0001**
Nutritional supplements, n (%)	1236 (38.1)	121 (46.5)		2703 (43.6)		575 (47.4)	992 (34.0)	306 (43.7)		0.17		**<0.0001**
Using nonpharmacological therapies, n (%)	943 (29.1)	71 (27.3)		2567 (41.4)		523 (43.1)	666 (22.8)	144 (20.5)		0.93		**<0.0001**

The group differences in the final 2 columns (main effect of hypertension [2 levels: normo, hyper] and main effect of HD status [3 levels: premanifest, manifest, and controls]) demonstrate the need for propensity score matching.

a Comorbidities other than essential hypertension.

### Hypertension in HD

BMI and ethnicity were included in the regression model (SMD > 0.1). The prevalence of essential hypertension was lower in HD patients (premanifest and manifest HD combined, 13.85%) compared with controls (19.34%; *z* = 5.60, *P* = 2.12 × 10^**−**8^). Independent of HD status, BMI (z = 20.40, *P* < 2 × 10^−16^) and ethnicity (χ^2^ = 16.0, *P* = 0.014) were associated with hypertension prevalence.

Examining risk factors for hypertension, we paradoxically found that HD patients consumed more units of alcohol (95% CI, 0.85–1.84 units per week; *P* = 1.23 × 10^−7^), smoked more cigarettes (95% CI, 0.91–2.22 cigarettes/day; *P* = 3.06 × 10^−6^), and had been smoking for more years (95% CI, 0.18–1.72 years; *P* = 0.015) compared with controls.

### Hypertension and Disease‐Onset Age

**Figure 1:**
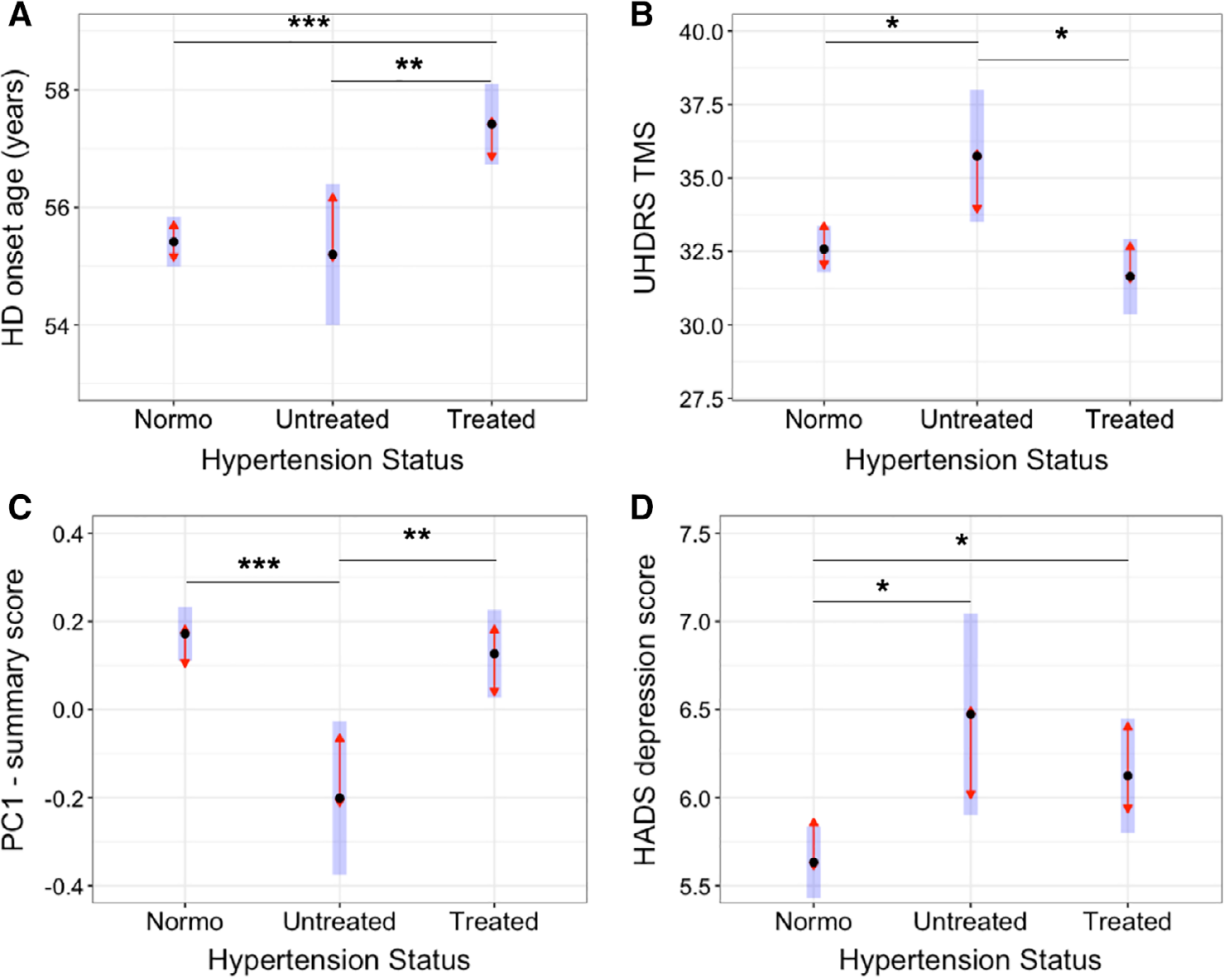
Effect of hypertension and associated antihypertensive treatment on HD clinical markers. Data shown are estimated marginal means (EMMs); black dot represents the mean, blue bars are 95% CIs for the EMMs, red arrows represent the Tukey‐based statistical comparison (overlapping arrows = notsignificant). ***p‐values < 0.001, **p‐values < 0.01, *p‐vales < 0.05. Normo = Normotensive, Untreated = Hypertensive patient not taking antihypertensive medication, PC1 = Cognitive summary statistic from the principal component analysis, principal component 1. [Color figure can be viewed at wileyonlinelibrary.com]

Normotensive HD patients were diagnosed with clinical onset an average of 1.5 years earlier than hypertensive HD patients (*F*_1,3402_ = 15.68, 95% CI, 0.75–2.21 years; *P* = 7.66 × 10^−5^). Follow‐up analyses comparing treated hypertensives (n = 908) and untreated hypertensives (n = 297) with normotensive HD patients (n = 2410) are shown in Figure 1A; treated hypertensives had an onset age 2.04 ± 0.41 years later than normotensives (*P* < 0.0001) and 2.25 ± 0.71 years later than untreated hypertensives (*P* = 0.004), whereas untreated hypertensives did not differ from normotensives (0.22 ± 0.65 years, *P* = 0.94).

### Hypertension and Motor Symptom Severity

**Figure 2:**
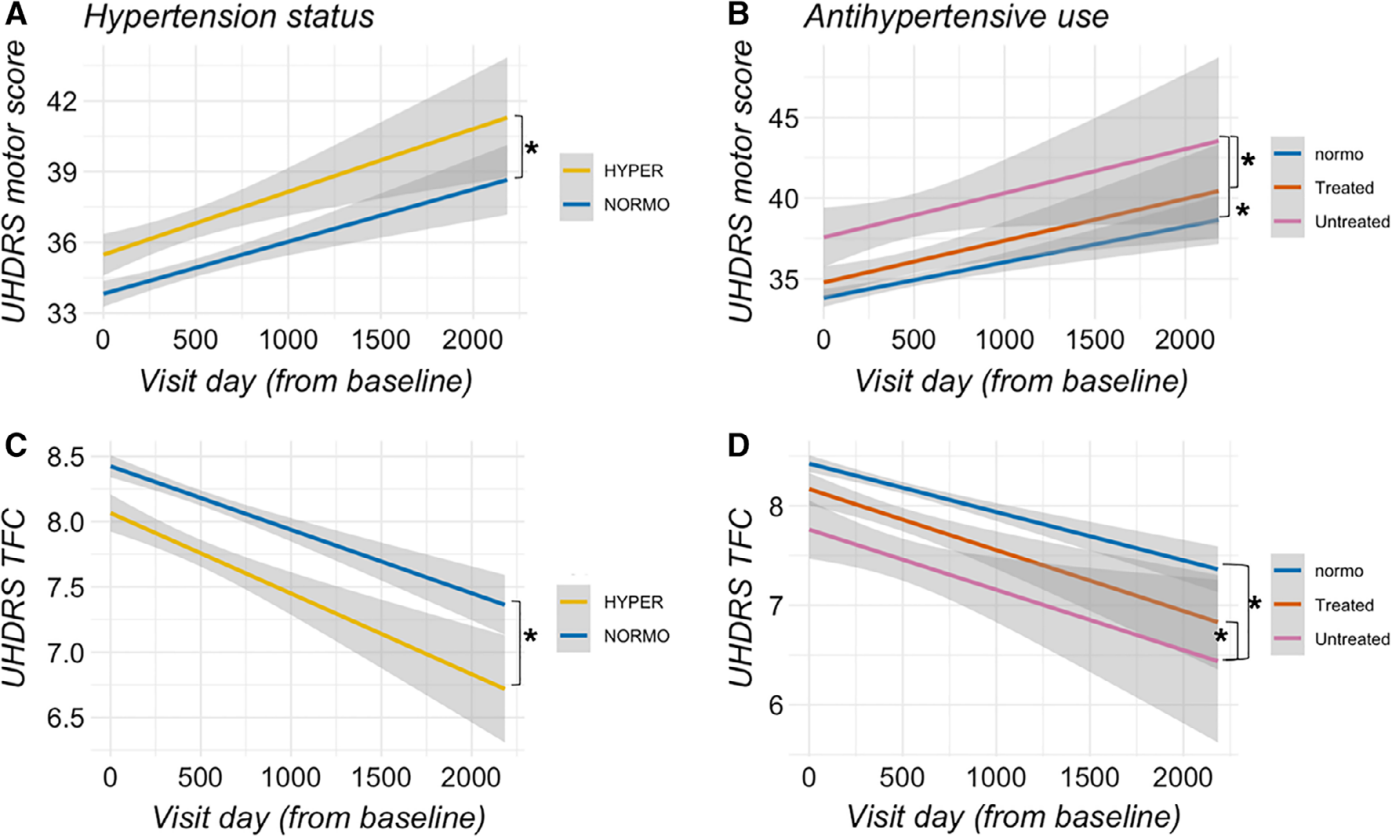
Disease progression in the motor (**A,B**) and functional domain (**C,D**) based on hypertension status (left panels) and treatment status (right panels). 95% confidence level interval for predictions from a linearmodel are displayed. *p < 0.05 from Tukey‐based statistical comparison. [Color figure can be viewed at wileyonlinelibrary.com]

A higher motor score is indicative of more motor impairment. At the baseline visit, there was no difference in motor score (TMS) between normotensives and hypertensives (*F*_1,4508_ = 0.76, *P* = 0.38; Fig. 1), whereas longitudinally, there was an interaction between time and hypertension status (95% CI, 0.00015–0.0011; *P* = 0.011; see Fig. 2A). Based on the findings related to HD disease onset, we analyzed TMS in premanifest HD and manifest HD participants separately and again did not find an association with hypertension (*P* = 0.35 and 0.12, respectively).

TMS was 3.51 ± 1.30 points higher in untreated hypertensive HD participants than in HD participants taking medication for hypertension (*P* = 0.019; see Fig. 1B) and 3.17 ± 1.19 points higher than in normotensives (*P* = 0.021), whereas there was no difference in motor score between treated hypertensives and normotensives (0.34 ± 0.776, *P* = 0.89).

The change in TMS over time was greater in untreated hypertensives (n = 372) compared with treated hypertensives (n = 1144, 2.88 ± 1.09, *P* = 0.02; Fig. 2B) and normotensives (n = 3032, 2.47 ± 0.99, *P* = 0.03) and did not differ between normotensive and treated hypertensive HD patients (*P* = 0.79). The duration of antihypertensive medication use did not predict motor score in treated hypertensive HD participants (*F*_1,1558_ = 0.16; 95% CI, −0.01 to 0.008; *P* = 0.69). Furthermore, for patients for whom HD onset occurred prior to hypertension diagnosis (n = 551), there was no difference in motor score between treated (n = 430) and untreated (n = 121) hypertensives (estimate = −1.11 ± 1.79; *P* = 0.93).

### Hypertension and Total Functional Capacity

A higher total functional capacity (TFC) is indicative of better functional ability. After matching, there was no difference in TFC between hypertensives and normotensives at baseline (*F*_1,4567_ = 0.11, *P* = 0.74). Longitudinally, there was an interaction between hypertension and time (95% CI, −1.02 to −0.0002; *P* = 0.029; Fig. 2C). Baseline TFC was lower in untreated hypertensive HD patients compared with treated hypertensive HD patients (0.61 ± 0.22 points, *P* = 0.02) and with normotensives (0.50 ± 0.20, *P* = 0.04), with no difference in TFC between treated hypertensives and normotensives (*P* = 0.67).

Longitudinally, untreated hypertensives had a steeper decline in TFC compared with treated hypertensives (−0.48 ± 0.19, *P* = 0.031) and normotensives (−0.43 ± 0.17, *P* = 0.034), with no difference between normotensive and treated hypertensives (*P* = 0.83, see Fig. 2D).

### Hypertension and Cognition

At baseline, hypertension had a significant effect on the cognitive summary score represented by PC1 (*F*_1,4575_ = 6.66; 95% CI, −0.24 to −0.03; *P* = 0.010), whereas there was no effect of hypertension on PC1 over time (*P* = 0.24). At baseline, hypertensive HD patients performed worse on the Letter Verbal Fluency and Trail Making (part B) tests (FDR‐adjusted *P* = 0.017 and 0.015, respectively). There was an interaction between hypertension status and time for performance on the Trail Making A subtest (95% CI, 0.005–0.002 seconds; *P* = 0.0017 FDR‐adjusted).

Antihypertensive treatment was associated with PC1 (*F*_1,4574_ = 8.34, *P* = 0.0002; see Fig. 1C) and all the cognitive subdomain tests, except for the Stroop Interference test (see Table 3). Untreated hypertensives had a lower PC1 score compared with normotensives (*P* = 0.0001) and treated hypertensives (*P* = 0.005), whereas the PC1 score was similar for treated hypertensive patients and normotensives (*P* = 0.57). Compared with normotensives and treated hypertensive patients, untreated hypertensive patients performed worse on the Symbol Digit Modalities Test, the Semantic Fluency test, and the Stroop word reading and color‐naming subtests (all *P* < 0.05). Untreated hypertensives were also significantly slower on both versions of the Trail Making test compared with normotensives. In contrast, treated hypertensives performed at a level similar to normotensives on all tests (all *P* > 0.05) except the Letter Verbal Fluency test (*P* = 0.05).

**Table 3:** Cognitive subscores (estimated marginal mean ± standard error of the mean, accounting for age) for HD patients with and without a diagnosis of essential hypertension from the baseline Enroll visit and with and without antihypertensive medication

				Pairwise comparison (*P*)			
	Normotensive	Untreated hypertensive	Treated hypertensive		Normo‐untreated	Normo‐treated	Untreated‐treated
Symbol Digit	26.8 ± 0.27	23.5 ± 0.78	26.4 ± 0.44		**0.0002**	0.79	**0.003**
Letter Verbal Fluency	25.2 ± 0.32	23.1 ± 0.91	23.7 ± 0.52		0.088	**0.049**	0.83
Semantic Fluency	13.3 ± 0.12	12.3 ± 0.34	13.3 ± 0.19		**0.019**	0.999	**0.038**
Stroop Word Reading	55.6 ± 0.44	50.9 ± 1.25	55.9 ± 0.71		**0.001**	0.94	**0.002**
Stroop Colour Naming	45.6 ± 0.36	41.9 ± 1.03	45.5 ± 0.58		**0.003**	0.99	**0.008**
Stroop Interference	25.8 ± 0.25	24.5 ± 0.71	25.0 ± 0.40		0.20	0.21	0.81
Trail Making A (s)^a^	66.8 ± 1.14	75.8 ± 3.26	68.0 ± 1.83		**0.03**	0.83	0.10
Trail Making B (s)^a^	135 ± 1.59	148 ± 4.54	141 ± 2.56		**0.01**	0.06	0.36

Normo, normotensive.

Boldface *P* values represent those that are statistically significant.

a A high time score on the Trail‐Making tests represents worse performance.

Over time, performance on Trail Making A and B differed between normotensive HD participants and untreated hypertensives (A, −8.68 ± 2.96; *P* = 0.009; B, −14.43 ± 3.81; FDR‐adjusted *P* = 0.004; Fig. 2F), and between normotensives and treated HD participants on subtest A (−6.49 ± 2.38, *P* = 0.018; Fig. 2F), whereas there was no difference between treated and untreated hypertensives (all *P* > 0.05). For PC1, there was a difference between normotensives and untreated hypertensives (0.29 ± 0.09, *P* = 0.003) and between treated and untreated hypertensives (0.24 ± 0.10, *P* = 0.034), yet no difference between treated hypertensives and normotensives (*P* = 0.68).

### Hypertension and Depression in HD

Hypertensive HD patients had a higher depression score (*F*_1,2585_ = 13.66; 95% CI, 0.31–1.0; *P* = 0.0002) and higher anxiety score (*F*_1,2580_ = 9.52; 95% CI, 0.19–0.87; *P* = 0.002) compared with normotensive HD patients. For 7.7% of hypertensive HD participants, there was a comorbid diagnosis of ongoing recurrent depression compared with 5.5% in normotensive HD participants (age‐adjusted odds ratio, 1.44; 95% CI, 1.12–1.83; *P* = 0.004).

Normotensive HD participants had a lower depression and anxiety score compared with treated hypertensive HD participants (depression: 95% CI, −0.18 to −0.94; *P* = 0.01; anxiety: 95% CI, −0.12 to −0.87; *P* = 0.02; Fig. 1D) and a lower depression score compared with untreated hypertensives (95% CI, −0.32 to −1.53; *P* = 0.008). There was no difference between treated and untreated hypertensive HD participants for depression and anxiety scores (*P* = 0.52 and 0.89, respectively) and no difference in anxiety scores between normotensive and untreated hypertensives (*P* = 0.08).

There was no interaction between hypertension and time for depression scores (95% CI, −0.0003 to 0.0002; *P* = 0.23; data not shown) or for anxiety scores over time (95% CI, −3.88 × 10^−5^ to 4.48 × 10^−4^; *P* = 0.10). Similarly, antihypertensives were not associated with depression or anxiety scores over time (*P* = 0.22 and 0.08, respectively, data not shown).

### Antihypertensive Medication

We compared hypertensive HD patients prescribed angiotensin‐converting enzyme inhibitors (31.0%), angiotensin receptor blockers (15.7%), beta‐blocking agents (18.0%), calcium channel blockers (17.2%), and diuretics (11.7%). The type of antihypertensive class had no effect on motor score (*P* = 0.68), age at onset (*P* = 0.26), TFC (*P* = 0.10), depression score (*P* = 0.78), anxiety score (*P* = 0.32), or performance on any cognitive test (all *P* > 0.05 FDR‐adjusted). Estimated marginal means are shown in Supplementary Figure 2.

## Discussion

Using rich longitudinal data from the largest observation study in HD, we present novel insights into the association between hypertension — a cardiovascular risk factor implicated in neurodegeneration — and HD disease severity and progression. Hypertension was detrimentally associated with HD disease severity in the cognitive and psychiatric domain and with motor symptom progression over time. Paradoxically, hypertension was also associated with a significant delay in the age at clinical HD onset in hypertensive patients. These observed differences between normotensive and hypertensive HD patients appear to be driven by antihypertensive medication use; hypertensive HD patients not receiving antihypertensive treatment had worse motor, cognitive, and functional capacity and more marked symptom progression over time compared with normotensive HD patients and hypertensive patients treated with antihypertensive medication, with potential implications for the clinical management of hypertension in HD.

The prevalence of hypertension was lower in HD patients despite HD patients consuming more alcohol and tobacco compared with age‐ and sex‐matched controls, both of which are risk factors for developing hypertension. This novel finding may be driven by differences in body mass between the groups; weight loss is a clinical feature of HD, and HD participants had a lower body mass index (BMI) than controls, despite propensity matching. In support of this, BMI was independently associated with hypertension prevalence. An earlier age at death in HD patients may have contributed to the finding, with fewer participants reaching the age at which hypertension develops. Alternatively, it may suggest that mutant Huntingtin has a protective effect on blood pressure homeostasis, plausibly a consequence of poor sympathoexcitatory pathways and poor autonomic control. This is in agreement with previous findings of orthostatic dizziness and a decreased orthostatic ratio in patients with moderate to severe HD.^20^

The paradoxical finding of a later age at clinic onset in hypertensive HD patients has been reported previously in a European subset cohort (n = 630) of the current worldwide data set^32^; however, this study did not statistically account for the confounding effects of age, sex, and BMI, which affect hypertension prevalence and HD disease progression.^34^, ^43^, ^44^, ^45^ Here we replicated the finding in this larger worldwide data set and across a wide distribution of CAG lengths while controlling for confounds. Crucially, we also showed that the delay in onset age was only associated with hypertensives taking antihypertensive medication, whereas untreated hypertensives had an age at disease onset similar to normotensives.

One interpretation of these results is that either antihypertensive medication, or the lowering of blood pressure is driving the delay in onset age, with a currently unknown mechanism and implications for HD management. Alternatively, the increasing prevalence of hypertension with increasing age may be biasing the data. For example, a patient with early HD onset is more likely to have faster disease progression and die earlier than a patient with later disease onset, which means that the normotensive group is skewed toward an earlier age at onset, with early‐onset patients less likely to live sufficiently long to develop hypertension. However, this does not explain the difference in HD onset age between treated and untreated hypertensives, for whom there was no difference in age prior to propensity matching. Furthermore, there was no difference in the frequency of death during the course of the longitudinal study between normotensive and hypertensive HD participants, suggesting that bias in the data is a less likely explanation.

Previous work, albeit equivocal, suggests that antihypertensive medication may bestow a neuroprotective effect for neurodegeneration, with evidence in Alzheimer's and Parkinson's diseases,^46^, ^47^, ^48^ whereas to the best of our knowledge the current study is the first to examine antihypertensive medication use in HD patients. Hypertensive HD patients who were not taking antihypertensive medication had more motor, cognitive, and functional impairment and an earlier age at clinical HD onset than hypertensive patients receiving treatment to control their blood pressure, whereas treated hypertensive patients had disease scores similar to normotensives. Increasing evidence shows that the cerebrovasculature is perturbed in HD^4^, ^26^, ^49^; thus, the detrimental effect of uncontrolled hypertension in HD may be because of a loading effect of hypertension‐induced cerebrovascular pathology on a weakened vascular system, with further work necessary to test this hypothesis.

Intriguingly, hypertensive patients with HD were prescribed a variety of different medications acting on different pathways. Angiotensin receptor blockers and calcium channel blockers are common antihypertensive treatments shown to have neuroprotective effects.^50^, ^51^, ^52^, ^53^ Here, we found no difference in onset age, motor score, or functional capacity between hypertensive HD patients prescribed different classes of antihypertensive medication, although a lack of sensitivity of these measures to neuropathology could explain this. Furthermore, we did not have the statistical power to account for pharmacological interactions between antihypertensives and other therapies used to manage the myriad evolving symptoms over the disease course of HD, yet this will be an important consideration for translation to clinical practice.

Mechanistically, it is not clear whether the detrimental effect of uncontrolled high blood pressure or the protective effect of antihypertensive medication is driving our findings, or their combination. The duration of antihypertensive medication use was not related to motor score, which may suggest that the reduced motor impairment observed in the treated hypertensives was driven by a reduction in high blood pressure. However, a shortcoming of this study was the lack of blood pressure measurements to determine if the antihypertensive medication controlled blood pressure. Crucially, these results identified an association between hypertension, antihypertensives, and HD disease severity, progression, and onset yet did not allow inference about causality. Further work should focus on establishing if a causal relationship exists, given the current lack of disease‐modifying therapies currently available for HD.

In conclusion, antihypertensive medication was associated with reduced disease severity for all clinical measures in hypertensive HD patients. Further investigation into the therapeutic efficacy of antihypertensive medication in cases of prehypertension in HD and in premanifest HD is warranted, along with the combinatorial effect with other HD symptom management therapeutics. This represents an exciting future avenue to explore the repurposing of specific antihypertensive drugs for the treatment of neurodegenerative disease.

## Author Contributions

Research Project: (A) Conception and design of the study: J.J.S., E.H., (B) Execution: J.J.S. 2) Statistical analysis: (A) Design: J.J.S., K.M., (B) Execution: J.J.S. 3) Manuscript: [A] Writing first draft: J.J.S., A.E.R., E.H., K.M., [B] Review and Critique: J.J.S., A.E.R., E.H., K.M.

## Financial Disclosures

Prof. Murphy and Dr. Steventon are funded by the Wellcome Trust (Senior Research Fellowship 200804/Z/16/Z). All other authors have tenure funding from their institutions.

## Supporting information


**Appendix** **S1:** Supplementary Data
