# Supplementary material for: Hypertension, Antihypertensive Use and the Delayed‐Onset of Huntington's Disease
Source: Mov Disord. 2020 Feb 4;35(6):937–46. doi: 10.1002/mds.27976 (PMC7317197; doi:10.1002/mds.27976)
Supplement: Supplementary file 1 — Appendix S1: Supplementary Data [file MDS-35-937-s001.docx]

# Supplementary Data

## Cognitive assessments

Verbal fluency examined the ability to spontaneously produce words orally within 60 seconds. For category fluency, words are produced according to semantic constraints. The SDMT involves a simple substitution task. Using a reference key, the participant had 90 seconds to pair specific numbers with given geometric figures. The Stroop Colour and Word Reading tests involve naming colours (e.g., red, green, blue) and reading the words for colours in black ink. Trail Making tasks are thought to reflect a wide variety of cognitive processes including visual attention, speed of processing, mental flexibility, and executive functions.

## Additional Analyses

Additional analyses examined the relationship between manifest HD disease duration (time since onset age) and hypertension duration and found no significant relationship (r=0.06, 95% CI [-0.006-0.131], p=0.076).

## ATC coding for antihypertensive medication

The following ATC codes were used for stratifying antihypertensives:

- Angiotensin converting enzyme inhibitors: C09AA
- Angiotensin receptor blockers: C09CA
- Calcium channel blockers: C08CA; C08CX; C08DA; C08DB; C08EA; C08EX
- Beta blocking agents: C07AA; C07AB
- Diuretics: C03AA; C03AB; C03BA; C03BB; C03BC; C03BD; C03BX; C03CA; C03CB; C03CC; C03CD; C03CX

Supplementary Table 1 Demographics after propensity matching based on antihypertensive medication use for age of onset analysis (manifest HD only included).

|  |  | **Age of onset** | | | | | | |
| --- | --- | --- | --- | --- | --- | --- | --- | --- |
|  |  | **Group count / mean** | | |  | **p-value** | | |
|  |  | **Normo (n=2426)** | **Treated hyper (n=916)** | **Untreated hyper (n=297)** |  | **Untreated vs. Treated** | **Untreated vs. Normo** | **Treated vs.Normo** |
| **Ethnicity (n, %)** | | | | |  | 0.57 | 0.32 | 0.84 |
| Caucasian |  | 2297 (94.7) | 863 (94.2) | 283 (95.3) |  |  | | |
| American Black |  | 19 (0.8) | 10 (1.1) | 2 (0.7) |  |  |  |  |
| Hispanic / Latino |  | 36 (1.5) | 16 (1.7) | 7 (2.4) |  |  |  |  |
| American Indian |  | 44 (1.8) | 13 (1.4) | 4 (1.3) |  |  |  |  |
| Asian |  | 6 (0.2) | 3 (0.3) | 0 (0) |  |  |  |  |
| Mixed |  | 15 (0.6) | 7 (0.8) | 1 (0.3) |  |  |  |  |
| Other |  | 9 (0.4) | 4 (0.4) | 0 (0) |  |  |  |  |
| **ISCED education level, n (%)** | | | | | | 0.25 | 0.47 | 0.12 |
| 0 |  | 16 (0.7) | 2 (0.2) | 1 (0.3) |  |  | | |
| 1 |  | 145 (6.0) | 55 (6.0) | 23 (7.7) |  |  |  |  |
| 2 |  | 448 (18.6) | 178 (19.6) | 61 (20.5) |  |  |  |  |
| 3 |  | 755 (31.3) | 339 (37.3) | 100 (33.7) |  |  |  |  |
| 4 |  | 406 (16.8) | 137 (15.1) | 44 (14.8) |  |  |  |  |
| 5 |  | 585 (24.2) | 180 (19.8) | 65 (21.9) |  |  |  |  |
| 6 |  | 59 (2.4) | 19 (2.1) | 3 (1.0) |  |  |  |  |
| **Employment status, n (% employed)** |  | 359 (14.8) | 137 (15.0) | 43 (14.5) |  | 0.26 | 0.58 | 0.11 |
| **Residence** | | | | |  | 0.21 | 0.10 | 0.75 |
| rural |  | 125 (5.2) | 49 (5.3) | 24 (8.1) |  |  | | |
| village |  | 461 (19.0) | 148 (16.2) | 40 (13.5) |  |  |  |  |
| town |  | 919 (37.9) | 349 (38.1) | 127 (42.8) |  |  |  |  |
| city |  | 917 (37.8) | 370 (40.4) | 106 (35.7) |  |  |  |  |
| **Marital Status** | | | | |  | 0.18 | 0.59 | 0.10 |
| single |  | 239 (9.9) | 72 (7.9) | 32 (10.8) |  |  | | |
| married |  | 111 (4.6) | 35 (3.8) | 12 (4.0) |  |  |  |  |
| partnership |  | 1575 (64.9) | 600 (65.5) | 184 (62.0) |  |  |  |  |
| divorced |  | 318 (13.1) | 121 (13.2) | 40 (13.5) |  |  |  |  |
| widowed |  | 143 (5.9) | 73 (8.0) | 23 (7.7) |  |  |  |  |
| legally separated |  | 35 (1.4) | 14 (1.5) | 6 (2.0) |  |  |  |  |
| **Comorbidities/concomitant** | | | | |  |  |  |  |
| Comorbidity, n (%) |  | 2209 (91.1) | 872 (95.2) | 282 (95.0) |  | ***0.0002*** | ***0.029*** | ***0.0002*** |
| Nutritional supplements, n (%) |  | 1126 (46.4) | 425 (46.4) | 150 (50.5) |  | 0.33 | 0.17 | 0.87 |
| Nonpharmacological therapies, n (%) |  | 1006 (41.5) | 394 (43.0) | 129 (43.4) |  | 0.82 | 0.45 | 0.65 |
| Current alcohol use, n (% yes) |  | 947 (39.1) | 338 (36.9) | 98 (33.0) |  | 0.17 | 0.07 | 0.50 |
| Alcohol units/week (SD) |  | 7.7 (9.9) | 7.6 (10.1) | 7.6 (12.3) |  | 0.998 | 0.94 | 0.89 |
| Current tobacco use, n (% yes) |  | 451 (18.6) | 182 (19.9) | 70 (23.6) |  | 0.18 | 0.06 | 0.56 |
| Tobacco pack years (SD) |  | 28.7 (19.8) | 26.1 (20.5) | 27.2 (22.9) |  | 0.93 | 0.89 | 0.42 |
| Current drug abuse, n (% yes) |  | 35 (1.4) | 12 (1.3) | 6 (2.0) |  | 0.68 | 0.61 | 0.54 |

Supplementary Table 2 Demographics after propensity matching based on antihypertensive medication use for disease severity analysis (premanifest and manifest HD combined).

|  |  | **Disease severity (premanifest and manifest HD)** | | | | | | |
| --- | --- | --- | --- | --- | --- | --- | --- | --- |
|  |  | **Normo (n=3032)** | **Treated hyper (n=1144)** | **Untreated hyper (n=372)** |  | **Untreated vs. Treated** | **Untreated vs. Normo** | **Treated vs.Normo** |
| **Ethnicity (n, %)** | | | | |  | 0.35 | 0.99 | 0.15 |
| Caucasian |  | 2885 (95.2) | 1076 (94.1) | 354(95.2) |  |  | | |
| American Black |  | 14 (0.5) | 12 (1.0) | 3 (0.8) |  |  |  |  |
| Hispanic / Latino |  | 50 (1.6) | 19 (1.7) | 7 (1.9) |  |  |  |  |
| American Indian |  | 48 (1.6) | 19 (1.7) | 5 (1.3) |  |  |  |  |
| Asian |  | 5 (0.2) | 4 (0.3) | 0 |  |  |  |  |
| Mixed |  | 21 (0.7) | 7 (0.6) | 2 (0.5) |  |  |  |  |
| Other |  | 9 (0.3) | 7 (0.6) | 1 (0.3) |  |  |  |  |
| **ISCED education level, n (%)** | | | | | | 0.25 | 0.47 | 0.12 |
| 0 |  | 16 (0.5) | 2 (0.2) | 1 (0.3) |  |  | | |
| 1 |  | 155 (5.1) | 56 (4.9) | 27 (7.3) |  |  |  |  |
| 2 |  | 553 (18.2) | 208 (18.2) | 72 (19.4) |  |  |  |  |
| 3 |  | 885 (29.2) | 407 (35.6) | 120 (32.3) |  |  |  |  |
| 4 |  | 557 (18.4) | 183 (16.0) | 61 (16.4) |  |  |  |  |
| 5 |  | 773 (25.5 | 259 (22.6) | 86 (23.1) |  |  |  |  |
| 6 |  | 76 (2.5) |  | 4 (1.1) |  |  |  |  |
| **Employment status, n (% employed)** |  | 774 (25.5) | 277 (24.2) | 91 (24.5) |  | 0.12 | 0.57 | ***0.04*** |
| **Residence** | | | | |  | ***0.03*** | ***0.01*** | 0.82 |
| rural |  | 170 (5.6) | 62 (5.4) | 33 (8.9) |  |  | | |
| village |  | 566 (18.7) | 187 (16.3) | 51 (13.7) |  |  |  |  |
| town |  | 1158 (38.2) | 429 (37.5) | 155 (41.7) |  |  |  |  |
| city |  | 1133 (37.4) | 466 (40.7) | 132 (35.5) |  |  |  |  |
| **Marital Status** | | | | |  | 0.2 | 0.43 | 0.15 |
| single |  | 310 (10.2) | 100 (8.7) | 43 (11.6) |  |  | | |
| married |  | 172 (5.7) | 58 (5.1) | 20 (5.4) |  |  |  |  |
| partnership |  | 1973 (65.1) | 739 (64.6) | 225 (60.5) |  |  |  |  |
| divorced |  | 377 (12.4) | 144 (12.6) | 51 (13.7) |  |  |  |  |
| widowed |  | 155 (5.1) | 84 (7.3) | 24 (6.5) |  |  |  |  |
| legally separated |  | 38 (1.3) | 18 (1.6) | 7 (1.9) |  |  |  |  |
| **Comorbidities/concomitant** | | | | |  |  |  |  |
| Comorbidity, n (%) |  | 2751 (90.7) | 1085 (94.8) | 352 (94.6) |  | ***1.50E-05*** | ***0.01*** | ***1.90E-05*** |
| Nutritional supplements, n (%) |  | 1369 (45.2) | 538 (47.0) | 177 (47.6) |  | 0.43 | 0.38 | 0.28 |
| Nonpharmacological therapies, n (%) |  | 1170 (38.6) | 460 (40.2) | 151 (40.6) |  | 0.53 | 0.45 | 0.34 |
| Current alcohol use, n (% yes) |  | 1304 (43.1) | 465 (40.7) | 143 (38.5) |  | 0.13 | 0.1 | 0.17 |
| Alcohol units/week (SD) |  | 7.9 (10.1) | (7.6 (10.2) | 8.0 (11.7) |  | 0.93 | 1 | 0.86 |
| Current tobacco use, n (% yes) |  | 594 (19.6) | 223 (19.5) | 89 (23.9) |  | 0.11 | ***0.04*** | 0.85 |
| Tobacco pack years (SD) |  | 27.3 (19.9) | 25.7 (19.8) | 25.9 (21.7) |  | 1 | 0.79 | 0.58 |
| Current drug abuse, n (% yes) |  | 59 (1.9) | 17 (1.5) | 11 (3.0) |  | 0.11 | 0.17 | 0.19 |

Supplementary Table 3 Demographics after propensity matching based on antihypertensive medication use for longitudinal analysis. Logistical regression was used for categorical data. † Employment status is a summary measure which includes full‐time employed, part‐time employed and self employed

|  | |  | | **Disease progression (premanifest and manifest HD)** | | | | | | | | | | | | | |
| --- | --- | --- | --- | --- | --- | --- | --- | --- | --- | --- | --- | --- | --- | --- | --- | --- | --- |
|  | |  | | **Normo (n=4342)** | | **Treated hyper (n=1149)** | | **Untreated hyper (n=372)** | |  | | **Untreated vs. Treated** | | **Untreated vs. Normo** | | **Treated vs.Normo** | |
| **Ethnicity (n, %)** | | | | | | | | | |  | | 0.34 | | 0.21 | | 0.37 | |
| Caucasian | |  | | 4076 (93.9) | | 1081 (94.1) | | 354 (95.2) | |  | |  | | | | | |
| American Black | |  | | 35 (0.8) | | 12 (1.0) | | 3 (0.8) | |  | |  |  |  |  |  |  |
| Hispanic / Latino | |  | | 77 (1.8) | | 19 (1.7) | | 7 (1.9) | |  | |  |  |  |  |  |  |
| American Indian | |  | | 79 (1.8) | | 19 (1.7) | | 5 (1.3) | |  | |  |  |  |  |  |  |
| Asian | |  | | 12 (0.3) | | 4 (0.3) | | 0 | |  | |  |  |  |  |  |  |
| Mixed | |  | | 34 (0.8) | | 7 (0.6) | | 2 (0.5) | |  | |  |  |  |  |  |  |
| Other | |  | | 29 (0.7) | | 7 (0.6) | | 1 (0.3) | |  | |  |  |  |  |  |  |
| **ISCED education level, n (%)** | | | | | | | | | | | | 0.64 | | 0.41 | | 0.71 | |
| 0 | |  | | 15 (0.3) | | 2 (0.2) | | 1 (0.3) | |  | |  | | | | | |
| 1 | |  | | 182 (4.2) | | 56 (4.9) | | 27 (7.3) | |  | |  |  |  |  |  |  |
| 2 | |  | | 739 (17.0) | | 210 (18.3) | | 72 (19.4) | |  | |  |  |  |  |  |  |
| 3 | |  | | 1305 (30.1) | | 408 (35.5) | | 119 (32.0) | |  | |  |  |  |  |  |  |
| 4 | |  | | 785 (18.1) | | 183 (15.9) | | 61 (16.4) | |  | |  |  |  |  |  |  |
| 5 | |  | | 1182 (27.2) | | 261 (22.7) | | 87 (23.4) | |  | |  |  |  |  |  |  |
| 6 | |  | | 111 (2.6) | | 22 (1.9) | | 4 (1.1) | |  | |  |  |  |  |  |  |
| **Employment status, n (% employed)†** | |  | | 1384 (31.9) | | 278 (24.2) | | 91 (24.5) | |  | | **8.80E+15** | | **0.0003** | | **7.95E+14** | |
| *Full-time* | |  | | *987 (22.7)* | | *178 (15.5)* | | *63 (16.9)* | |  | |  | |  | |  | |
| *Part-time* | |  | | *307 (7.1)* | | *73 (6.4)* | | *18 (4.8)* | |  | |  | |  | |  | |
| *Self-employed* | |  | | *90 (2.1)* | | *27 (2.3)* | | *10 (2.7)* | |  | |  | |  | |  | |
| **Residence** | | | | | | | | | |  | | **0.004** | | **0.001** | | 0.8 | |
| rural | |  | | 252 (5.8) | | 62 (5.4) | | 33 (8.9) | |  | |  | | | | | |
| village | |  | | 778 (17.9) | | 186 (16.2) | | 51 (13.7) | |  | |  |  |  |  |  |  |
| town | |  | | 1633 (37.6) | | 430 (37.4) | | 155 (41.7) | |  | |  |  |  |  |  |  |
| city | |  | | 1672 (38.5) | | 471 (41.0) | | 132 (35.5) | |  | |  |  |  |  |  |  |
| **Marital Status** | | | | | | | | | |  | | **6.60E-10** | | 0.33 | | **8.43E-11** | |
| single | |  | | 576 (13.3) | | 100 (8.7) | | 43 (11.6) | |  | |  | | | | | |
| married | |  | | 276 (6.4) | | 58 (5.0) | | 19 (5.1) | |  | |  |  |  |  |  |  |
| partnership | |  | | 2717 (62.6) | | 742 (64.6) | | 226 (60.8) | |  | |  |  |  |  |  |  |
| divorced | |  | | 507 (11.7) | | 145 (12.6) | | 51 (13.7) | |  | |  |  |  |  |  |  |
| widowed | |  | | 181 (4.2) | | 85 (7.4) | | 24 (6.5) | |  | |  |  |  |  |  |  |
| legally separated | |  | | 78 (1.8) | | 18 (1.6) | | 7 (1.9) | |  | |  |  |  |  |  |  |
| **Comorbidities/concomitant** | | | | | | | | | |  | |  | |  | |  | |
| Comorbidity, n (%) | |  | | 3956 (91.1) | | 1090 (94.9) | | 352 (94.6) | |  | | ***2.20E-16*** | | ***2.15E-06*** | | ***2.77E-13*** | |
| Nutritional supplements, n (%) | |  | | 2037 (46.9) | | 541 (47.1) | | 178 (47.8) | |  | | ***0.001*** | | ***0.0002*** | | 0.69 | |
| Nonpharmacological therapies, n (%) | |  | | 1801 (41.5) | | 465 (40.5) | | 151 (40.6) | |  | | 0.37 | | 0.71 | | 0.19 | |
| Current alcohol use, n (% yes) | |  | | 1944 (44.8) | | 463 (40.3) | | 144 (38.8) | |  | | ***3.10E-07*** | | ***6.54E-06*** | | ***0.000331*** | |
| Alcohol units/week (SD) | |  | | 7.97 (10.2) | | 9.05 (10.2) | | 8.17 (11.7) | |  | | 0.25 | | 0.92 | | ***0.0008*** | |
| Current tobacco use, n (% yes) | |  | | 932 (21.5) | | 224 (19.5) | | 89 (23.9) | |  | | 0.24 | | 0.45 | | 0.11 | |
| Tobacco pack years (SD) | |  | | 24.7 (19.6) | | 25.7 (19.8) | | 25.9 (21.7) | |  | | 0.26 | | 0.03 | | 0.44 | |
| Current drug abuse, n (% yes) | |  | | 99 (2.3) | | 17 (1.5) | | 11 (3.0) | |  | | ***1.70E-05*** | | 0.91 | | ***2.66E-06*** | |


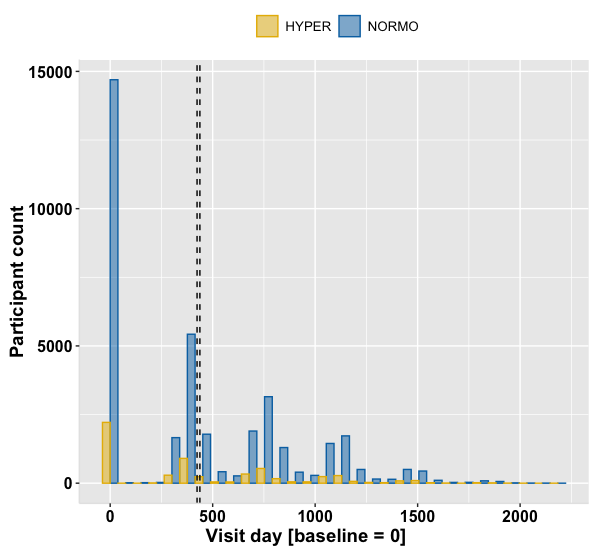


Supplementary Figure 1 Histogram showing the longitudinal follow up period and the participant numbers, stratified for hypertension status and prior to propensity matching.


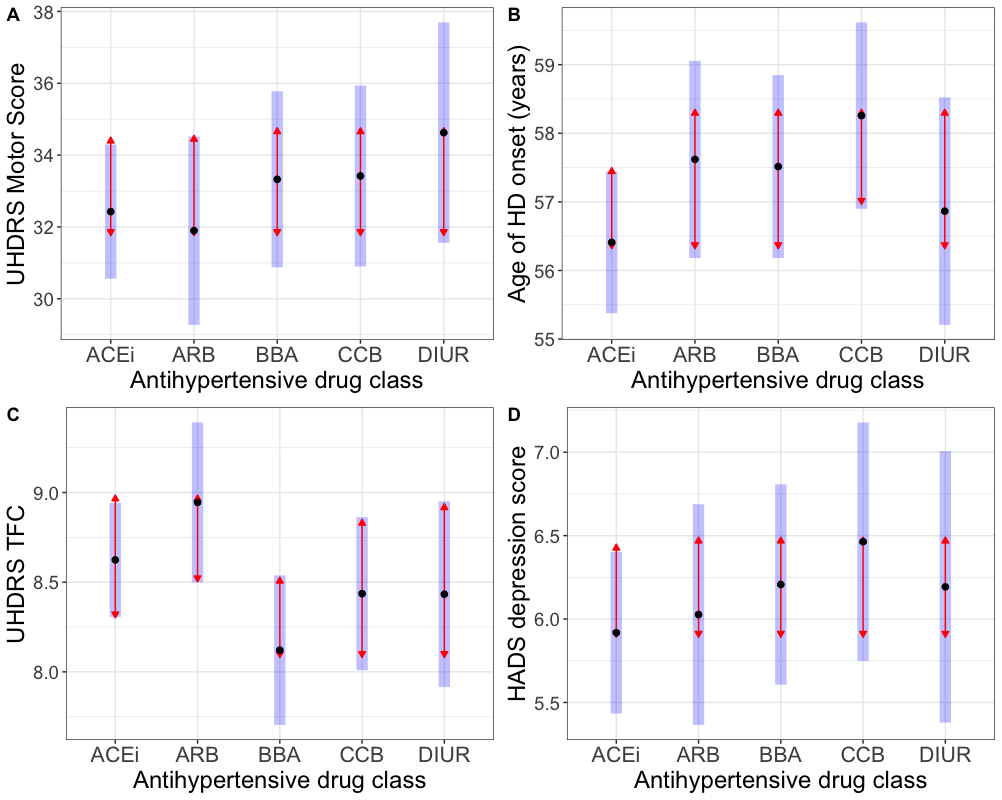


Supplementary Figure 2 Estimated marginal means (EMMs) from the linear model analyses, adjusted for sex, examining the effect of antihypertensive drug class on HD age of onset and clinical measures in hypertensive HD participants. Black dot represents the mean, blue bars are 95% CIs for the EMMs, red arrows represent the Tukey-based statistical comparison (overlapping arrows = not significant).
